# Detection of Pathogens of Acute Febrile Illness Using Polymerase Chain Reaction from Dried Blood Spots

**DOI:** 10.4269/ajtmh.21-0814

**Published:** 2021-11-08

**Authors:** Brian Grundy, Ursula Panzner, Jie Liu, Hyon Jin Jeon, Justin Im, Vera von Kalckreuth, Frank Konings, Gi Deok Pak, Ligia Maria Cruz Espinoza, Abdramane Soura Bassiahi, Nagla Gasmelseed, Raphaël Rakotozandrindrainy, Suzanne Stroup, Eric R. Houpt, Florian Marks

**Affiliations:** ^1^ Division of Infectious Diseases and International Health, University of Virginia, Charlottesville, Virginia;; ^2^ International Vaccine Institute, Seoul, Republic of Korea;; ^3^ Swiss Tropical and Public Health Institute, Basel, Switzerland;; ^4^ University of Basel, Basel, Switzerland;; ^5^ Cambridge Institute of Therapeutic Immunology and Infectious Disease, University of Cambridge School of Clinical Medicine, Cambridge Biomedical Campus, Cambridge, United Kingdom;; ^6^ Institut Supeìrieur des Sciences de la Population, University of Ouagadougou, Burkina Faso;; ^7^ Faculty of Medicine, University of Gezira, Wad Medani, Sudan;; ^8^ University of Antananarivo, Antananarivo, Madagascar

## Abstract

Quantitative polymerase chain reaction (qPCR) of dried blood spots (DBS) for pathogen detection is a potentially convenient method for infectious disease diagnosis. This study tested 115 DBS samples paired with whole blood specimens of children and adolescent from Burkina Faso, Sudan, and Madagascar by qPCR for a wide range of pathogens, including protozoans, helminths, fungi, bacteria, and viruses. *Plasmodium *spp. was consistently detected from DBS but yielded a mean cycle threshold (Ct) 5.7 ± 1.6 higher than that from whole blood samples. A DBS qPCR Ct cutoff of 27 yielded 94.1% sensitivity and 95.1% specificity against the whole blood qPCR cutoff of 21 that has been previously suggested for malaria diagnosis. For other pathogens investigated, DBS testing yielded a sensitivity of only 8.5% but a specificity of 98.6% compared with whole blood qPCR. In sum, direct PCR of DBS had reasonable performance for *Plasmodium* but requires further investigation for the other pathogens assessed in this study.

Acute febrile illness (AFI) is one of the most common reasons for patients to seek healthcare.^1^ There are many infectious agents, including bacteria, viruses, fungi, and parasites, that require a wide array of diagnostic methods.^2^ Quantitative polymerase chain reaction (qPCR) provides a sensitive and specific method for pathogen detection.^3^ However, PCR-based diagnostics are often unavailable in resource-limited settings; therefore, samples must be collected and tested later. Storing and shipping frozen samples can be costly and challenging. Dried blood spots (DBS) are a convenient alternative because only a small volume is required, and storage is typically at ambient temperature. DBS are of use for newborn HIV diagnosis^4^ and screening for sickle cell disease,^5^ among many other uses.^6^ Recent studies have shown the potential of molecular detection of pathogens using DBS including *Plasmodium *spp.,^7^^,^^8^ dengue fever virus,^9^ *Leptospira *spp.,^10^ *Streptococcus *spp.^11^ and *Haemophilus *spp.^11^ but with varying success.

The Typhoid Fever Surveillance in Africa Program (TSAP) was a multisite prospective fever surveillance study conducted in 10 sub-Saharan African countries between 2011 and 2013.^12^ The project’s main goal was to identify *Salmonella* Typhi and invasive nontyphoidal *Salmonella *spp. in febrile patients using blood culture-based diagnostics.^13^ In a recent investigation using whole blood samples from Sudan, Burkina Faso, and Madagascar, we identified by qPCR multiple pathogens not detected previously.^14^ Here, we compare the performance of qPCR on paired DBS.

A total of 615 whole blood samples from Burkina Faso, Sudan, and Madagascar, were stored at –80°C and tested in 2018 by qPCR by TaqMan Array Card (TAC).^14^ Of these 615 samples, we selected 107 DBS for which whole blood was positive for one or more of 15 pathogens, in addition to eight DBS for which blood was negative for all pathogens. DBS were prepared on Whatman FTA mini cards (WB120055, Cytiva, Buckinghamshire, UK) from venous blood at the time of enrollment in 2011 and 2013, stored at room temperature at the International Vaccine Institute, South Korea, and tested at the University of Virginia in 2021. The ethical clearance for the underlying TSAP program included additional pathogen identification including this work. The International Vaccine Institute’s Institutional Review Board (IRB), the Comiteì d’Ethique pour la Recherche en Santeì of the Ministry of Health in Burkina Faso, the Comiteì d’Ethique of the Ministry of Health of the Republic of Madagascar, and the National Research Ethics Review Committee of the National Ministry of Health in Sudan provided ethical approvals. The University of Virginia IRB provided additional approval for this work.

DBS were hole punched with a 3-mm Harris Uni-core hole puncher. Hole punchers were cleaned after use by soaking in 70% ethanol and Eliminase (VWR, Avantor, Radnor, PA). Nucleic acid extraction was carried out on six hole punches using the QIAamp DNA mini kit (Qiagen, Hilden, Germany) according to manufacturer’s instructions. Phocine herpesvirus 1 (PhHV-1; 10^6^ copies) and MS2 bacteriophage (10^7^ copies) were added to monitor extraction and amplification efficiency. Extraction blanks were included to monitor for laboratory contamination.

On the basis of the previous TAC results,^14^ cognate qPCR assays targeting *Aeromonas baumannii*, *Aeromonas *spp., *Bartonella *spp., cytomegalovirus, dengue virus, enterovirus, *Escherichia coli*, *Histoplasma *spp.,* K. oxytoca*, *M. tuberculosis*, *Plasmodium *spp., *Rickettsia *spp.,* Staphylococcus aureus, Streptococcus pneumoniae*, and *Schistosoma *spp., were performed on 96-well plates as individual reactions. Each assay target was tested on at least 23 samples each, including known positive samples for each target based on blood TAC results and randomly selected negative samples from all 115 extracted DBS. Each 20-µL reaction was carried out with 10 µL nucleic acid extract, 5 µL TaqMan Fast Virus master mix (Life Technologies, Thermo Fisher Scientific, Carlsbad, CA), 900 nM primers, and a 250-nM probe as described in the previous study.^14^ Cycling conditions (same as TAC) included reverse transcription for 10 minutes at 50°C, denaturation for 20 seconds at 95°C, and 40 PCR cycles of 3 seconds at 95°C and 30 seconds at 60°C. Nuclease-free water and a combined positive control template^15^ were used as controls.

A sample was considered positive if a reaction yielded a cycle threshold (Ct) of less than 40. DBS Ct-values were compared with whole blood TAC qPCR Ct-values to assess correlation and sensitivity and specificity. Statistical analysis, including frequencies, correlations, and receiver operator curve (ROC) analysis, was carried out using SPSS version 26.

*Plasmodium *spp. was tested in 95 DBS samples (Figure 1), of which 48 were positive in paired whole blood samples. Of the 48 paired DBS, 44 were positive for *Plasmodium *spp., yielding a DBS sensitivity of 91.7%. Of the 47 samples that were negative for *Plasmodium *spp. in whole blood, eight DBS were positive, with higher Cts ranging from 32.5 to 38.9, yielding a specificity of 83.0%. The median Ct for positive DBS samples for *Plasmodium *spp. was 23.9 (interquartile range [IQR]: 21.9–26.7) compared with a whole blood Ct median of 18.7 (IQR: 16.4–21.7) on TAC.^14^ The mean increase in DBS Ct for samples positive by whole blood for *Plasmodium *spp. was 5.7 ± 1.6. A whole blood qPCR Ct-value of 21 on TAC corresponds to a few thousand parasites per microliter, which has been used as a clinical case definition.^16^ Against this standard, the optimal DBS cutoff was 27 (ROC area under the curve: 0.98) and yielded a sensitivity and specificity of 94.1% and 95.1%, respectively.

**Figure 1.**
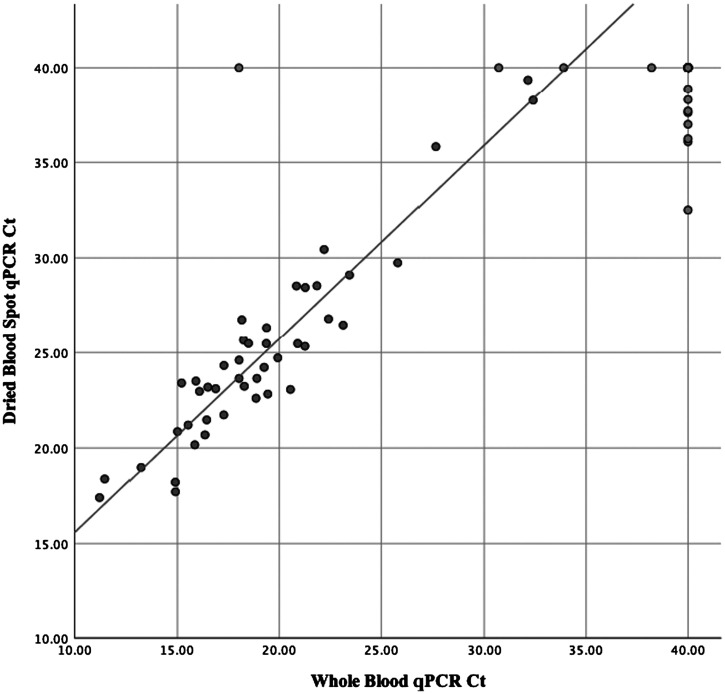
Correlation of whole blood specimens paired with dried blood spots by qPCR for *Plasmodium *spp. Line of best fit (y = 1.02x + 5.41, *R*^2^ = 0.889) of Ct-values, excluding negative values (Ct = 40). Ct = cycle threshold; DBS = dried blood spot; qPCR = quantitative polymerase chain reaction.

Beyond *Plasmodium *spp., there were 59 whole blood samples that were qPCR positive for one or more of 14 other pathogens. These whole blood Cts were generally high (median: 34.7, IQR: 33.6–36.3). The corresponding DBS were tested with the relevant qPCR assays, and 5/59 (1 *E. coli*, 2 cytomegalovirus, 1 *S. pneumoniae*, 1 *S. aureus*) were positive with Cts ranging from 35.3 to 39.5 leading to 8.5% sensitivity. Specificity of DBS by qPCR was assessed by performing myriad assays (as described in methods) on DBS with paired whole blood negative samples (total PCR reactions = 340), of which four (one cytomegalovirus, two dengue, one *E. coli*) were DBS PCR-positive with Cts ranging from 34.3 to 39.5, resulting in 98.6% specificity.

This work explored the capabilities of DBS for pathogen detection by qPCR. Although the detection of *Plasmodium *spp. from DBS correlated reasonably well with detection from whole blood, DBS qPCR was insensitive for the other pathogens of AFI that we evaluated.^14^ Other studies have shown promising results in *Plasmodium *spp. detection by DBS PCR and higher Cts compared with whole blood,^7^^,^^8^ but findings have been inconsistent for other pathogens.^9^^–^^11^ This is likely due to a higher quantity of malaria parasites in blood compared with that of bacteria and certain viruses.

This study was limited by small sample size for many pathogens, and we would also note the long duration of storage of the DBS. Ideally, DBS samples would be tested within a few days to several months.^8^^–^^10^ Despite this limitation, pathogen DNA was still detected more than 8 years later, especially for *Plasmodium *spp. Others have also found amplification of DNA from filter paper after 7 years despite storage at room temperature.^17^ Nonetheless, given the small volume of blood applied onto DBS and the possibility of nucleic acid degradation over time, particularly RNA, studies are needed to evaluate the use of new filter paper products, extraction techniques, and storage conditions. Another observation we noted during pilot testing of purification and amplification methods was that contamination from hole punchers used in DBS analyses is a potential risk,^18^^–^^20^ especially for *Plasmodium *spp. detection. We tested and developed a new cleaning method of hole punchers to minimize the risk of contamination. In sum, direct PCR of DBS has good performance for clinical malaria and could be useful for malaria surveillance but requires further investigation for the other pathogens assessed in this study.
